# Occult Hepatitis B Infection and its Possible Impact on Chronic Hepatitis C Virus Infection

**DOI:** 10.4103/1319-3767.56089

**Published:** 2009-10

**Authors:** Peiman Habibollahi, Saeid Safari, Nasser E. Daryani, Seyed M. Alavian

**Affiliations:** 1,2 Gastroenterology Division, Tehran University of Medical Sciences, Tehran, Iran; 3 Department of Internal Medicine, Tehran University of Medical Sciences, Tehran, Iran; 4 Baqiyatallah Research Center for Gastroenterology and Liver Diseases, Baqiyatallah University of Medical Sciences, Tehran, Iran

**Keywords:** Hepatitis B, hepatitis C, occult

## Abstract

As a well-recognized clinical phenomenon, persistent detectable viral genome in liver or sera in the absence of other serological markers for active hepatitis B virus (HBV) replication is called occult HBV infection. The main mechanism through which occult infection occurs is not completely understood and several possible explanations, such as integration into human genome and maintenance in peripheral mononuclear cells, exist. Occult HBV infection has been reported in different populations, especially among patients with Hepatitis C (HCV) related liver disease. The probable impact of occult HBV in patients with chronic HCV infection has been previously investigated and the evidence suggests a possible correlation with lower response to anti-viral treatment, higher grades of liver histological changes, and also developing hepatocellular carcinoma. However, in the absence of conclusive results, further studies should be conducted to absolutely assess the impact of occult HBV contamination on the HCV related liver disease.

Occult infection with hepatitis B virus (HBV), defined as persistent detectable viral genome in serum or liver while HBV surface antigen (HBsAg) is undetectable, is not a novel concept and has been described since the early 1980's.[1] The description follows the development of highly sensitive diagnostic tests able to detect minute quantities of HBV genome.[1–3] However, several other explanations for this condition, such as HBV DNA sequence alteration by mutation[4] and viral DNA integration in the host genome,[5] have also been suggested.

More recently, occult HBV infection has been considered to play a role in some clinical phenomena including cryptogenic liver disease,[6] and a poor response to antiviral treatment[7] and development of hepatocellular carcinoma[8] in chronic hepatitis C virus (HCV) infected patients. In addition, chronic HCV infection, due to almost the same route of transmission, is frequently reported with occult HBV infection.[9–12]

Considering the implications of occult HBV infection and the growing evidence on this subject, we have aimed to review the available data and highlight the most important points, and it's role in response to antiviral treatment in patients with chronic HCV infection.

## HISTORY

In 1985, Brechot *et al.* reported detection of HBV Deoxyribonucleic acid (DNA) in serum or liver of patients with chronic liver disease. The authors concluded that HBV DNA multiplication might occur in the absence of any HBV conventional serologic markers.[1] This provided the trigger for a number of other studies to test the concept in other clinical situations. In 1993, persistent anti-HBc reactivity was suggested as the only sign of occult HBV infection in the absence of other serologic markers.[13] Occult HBV infection was also shown to serve as a source of post transplant HBV infection in patients who receive liver transplantation.[14] Other studies also confirmed the presence of viral genome in tissues other than liver.[15]

These studies emphasized the role of so called occult HBV infection in different conditions and also the active nature of disease as a transmissible infection. Based on these preliminary results, several other studies were planned. The presence of seronegative HBV infection in patients with chronic hepatitis due to HCV was frequently reported.[11,16] In 1999, Cacciola *et al.* studied the existence of HBV genome in patients with HCV related chronic hepatitis and reported a prevalence rate of about 33% among these patients.^2^ These findings suggested a coincidence for HBV and HCV infection and mentioned a possible role for occult HBV infection in the clinical and pathologic features of chronic HCV-related liver disease.

## PROBABLE MECHANISMS OF OCCULT INFECTION

There are several explanations for silent or undetectable HBV infection. The development of more sensitive and improved techniques for HBV DNA detection has led to introduction of occult HBV which was not traceable earlier.[17] This is similar to the increase in the prevalence of disease with improvement of diagnostic tools and seems to be correct at least in some cases, although other possible mechanisms have also been suggested.

HBV reactivation in immunosuppressed patients reveals a role for the host immune system in occult form of HBV infection. The immune system might keep the viral replication at very low levels which are not detectable by normal screening.[18–20] Likewise, hepatic cytokines such as TNF-α and IFN-β-may inhibit viral replication and activation.[21,22] Integration of viral genome in human DNA and mutations modifying the viral antigens are also among the other possible theories[4,5] but these mutations have not been shown in other studies.[2,10] Tamori *et al.* demonstrated HBV DNA integration in human genome and suggested a role for occult HBV in accelerating the hepatocarcinogenesis in chronic hepatitis due to HCV.[5] HBV variants according to the surface genes, and different response of the host to these variants are other possible presumptions for the presence of HBV DNA in HBsAg negative patients.[4]

Early viral genome integration in liver and peripheral mononuclear cells,[23] existence of immune complexes which contain HBV DNA[24] and modified immunological response in the patient[25] are among the other probable explanations. Some other studies also suggest that the presence of other viral infections such as HCV may interfere with HBV course and detection.[26–28] This hypothesis has been empowered by in vitro analysis which showed inhibition of HBV replication by NS2 and core HCV proteins.[29–32] The exact mechanism of occult infection with HBV is not fully understood, and it is feasible that the hypotheses mentioned above could all in part contribute in the course of the disease.

## OCCULT HBV IN HCV PATIENTS

Several studies have reported occult HBV infection in patients with HCV related disease. However, due to a heterogeneous study population, a net conclusion linking these results is not very straightforward. Table 1 briefly describes the results of the most important studies regarding occult HBV prevalence in HCV related liver disease. The prevalence of occult HBV in different studies has been reported between zero and 52.3% among patients with diverse liver disease due to HCV infection.[9,33,34]

**Table 1:** Characteristics of various studies on occult HBV in HCV-infected patients

Authors	Year	Target population	Occult HBV prevalence (%)	Comments
Cacciola[2]	1999	200 with chronic HCV-related liver disease 50 with unrelated to HCV	33 14	33 patients with occult HBV had cirrhosis compared to 19 precent without (*P* = 0.04)
Fukuda[22]	1999	66 with chronic HCV-related liver disease	52.3	Higher prevalence for genotype 1b than in 2a (64.3% vs 28.6%, *P* < 0.01)
Kao[21]	2002	210 with HCV-related liver disease 100 healthy controls	14.8 15	Study concluded that occult HBV has no significance in HCV-related liver disease
Besisik[23]	2003	33 HBsAg negative hemodyalitic patients with HCV-related liver disease	33.4	-
Giannini[24]	2003	119 with HCV-related liver disease	6.7	No difference in the presence of occult HBV infection was seen between various degrees of liver disease
Georgiadou[25]	2004	187 with HCV-related liver disease	26.2	HBV-DNA was neither associated with HBV markers, nor with the clinical status of HCV patients.
Khattab[26]	2005	53 HBsAg-negative patients with chronic hepatitis C	7.5	Study could not show any impact of occult HBV in these patients
Goral[27]	2006	50 HBsAg negative hemodyalitic patients with HCV-related liver disease	0	-
Branco[28]	2007	46 with HCV related liver disease	19.5	Occult HBV infection was much more in cases with hepatocellular carcinoma
Toyoda[29]	2007	95 patients with HCV related hepatocellular carcinoma	2.1	HBV infection does not appear to play an important role in hepatocarcinogenesis
Altindiş[30]	2007	40 HCV infected hemodyalitic patients	27.5	Higher rates of HBV infection in hemodialysis patients
		41 HCV infected non hemodyalitic patients	2.4	
Alencar[31]	2008	33 patients with HCV related cirrhosis 17 patients with HCV related hepatocellular carcinoma	0 5.8	Study showed higher prevalence of HCV genotype 3 among Brazilian patients with cirrhosis and hepatocellular carcinoma
Miura[7]	2008	141 patients with chronic HCV-related liver disease	5.6	Study showed HBV as a risk factor for hepatocellular carcinoma development in patients with HCV
Ramia S[32]	2008	98 HCV infected patients from different institutions 85 controls with anti-HBC antibody 85 healthy controls	16.3 41 7.1	As the severity of liver disease increases the rate of positivity for HBV DNA increases
Shetty[33]	2008	56 patients with HCV cirrhosis	50	Occult HBV is associated with hepatocellular carcinoma
Sagnelli[34]	2008	89 patients with biopsy proven chronic HCV	41.6	No association was found between occult HBV infection and the degree of liver necroinflammation and fibrosis

Several factors could be responsible for this dissimilarity among studies. The most important of all might be heterogeneity of study populations. Higher probability of acquisition for “at risk populations” such as patients undergoing dialysis for both HBV and HCV infection might lead to higher prevalence rates [35] compared to those who do not have such risk factors. A common source of transmission such as intravenous drug use is also another factor which can affect these rates.

In contrast, different techniques used to detect the HBV DNA have different sensitivities so it might lead to different and incomparable results.[36–39] Regional differences in original HBV and HCV prevalence are other important factors.

## SEVERITY AND HISTOLOGY OF HCV RELATED LIVER DISEASE

Several investigators have studied the relationship between severity of HCV-linked liver disease and concomitant occult HBV infection. Cacciola et al, reported a high percentage of cirrhotic patients with occult HBV and no association with chronic hepatitis.[2] In the same year, occult HBV was reported to be seen in patients with higher hepatitis activity index although this association could not reach a statistically significant level.[33]

Several other studies have shown that there is no correlation between clinical outcomes and severity of liver disease and silent HBV infection[28,38,40–43] although recent studies have contradicted earlier reports, and emphasized on the clinical impact of silent HBV in patients suffering from chronic liver disease as a result of HCV and reported that higher levels of histological changes and hepatocellular carcinoma are seen among these patients.[7,8,44] It should be taken into consideration that most of these studies are cross sectional and therefore more precise cohort studies have to be performed to measure the real impact of occult HBV in patients with HCV linked chronic liver disease.[18]

## OCCULT HBV AND HCV GENOTYPES

Some studies have shown higher prevalence of occult HBV infection in HCV genotype 1b infected patients[33] compared to HCV genotype 2a. Following these findings most other studies failed to demonstrate such relationship between the HCV genotypes and occurrence of occult HBV infection.[7,28,40,41,43,45]

## SERUM AMINOTRANSFERASES

A relationship between the associated silent HBV and chronic HCV disease and high aminotransferases levels has been suggested by preliminary studies. Fukuda *et al*, found higher serum alanine aminotransferase levels in occult HBV-infected patients although, the study failed to demonstrate a significant correlation.[33]

To the best of our knowledge, almost none of the studies report a relationship between silent HBV infection and high aminotransferases levels in patients suffering from chronic liver disease due to HCV infection[7,28,40,41,44] and it can be concluded that aminotransferase level in patients with chronic HCV infection cannot predict the presence of silent HBV replication or activation.

## RESPONSE TO ANTIVIRAL TREATMENT

Treatment failure in chronically infected patients with HCV has been investigated widely due to its high priority among contemporary health problems. Several factors as well as concomitant occult HBV infection were tested; the diverse results obtained throughout the studies, make it impossible to come to a convincing conclusion about the role of silent HBV. Preliminary studies showed a trend towards weaker response to interferon mono-therapy in the presence of occult HBV.[2,33] However, these were followed by several studies which differed in their views and emphasized a minimal role for occult HBV in occult contamination in response to interferon mono-therapy or its combination with ribavirin.[28,40–43,45]

Due to the differing results obtained in various studies, and the absence of prospective controlled trials, it becomes imperative to perform such trials to clarify the role of occult HBV infection and its impact on anti-HCV treatment. There is dissimilarity in results obtained and no prospective controlled investigations. Hence it is of utmost importance to perform such investigations to clarify the role of occult HBV infection in response to anti-HCV treatment.

## CONCLUSION

In conclusion, available data, concerning the impact and prevalence of occult HBV infection in chronically infected patients with HCV, points to a considerable concomitance which may accompany higher grades of liver involvement and poorer response to anti-viral treatment. However, due to a scarcity of well-designed cohort studies, adequate conclusions can only be arrived at following further research on the subject.
